# Determination of gaps in the spatial accessibility of nursing services for persons over the age of 65 with lowered self-sufficiency: Evidence from the Czech Republic

**DOI:** 10.1371/journal.pone.0244991

**Published:** 2021-01-11

**Authors:** Iveta Vrabková, Izabela Ertingerová, Pavel Kukuliač

**Affiliations:** 1 Faculty of Economics, Department Public Economics, VŠB-TU Ostrava, Czech Republic; 2 Faculty of Mining and Geology, Department of Geoinformatics, VŠB-TU Ostrava, Czech Republic; University of Defence in Belgrade, SERBIA

## Abstract

The subject of this research is one of the main preconditions for the provision of high-quality social care services for people over the age of 65 with lowered self-sufficiency. It involves the spatial accessibility of formally established nursing services examined in 76 districts of the Czech Republic. The aim of this article is to identify and evaluate the gaps in spatial accessibility of the selected residential and outpatient-clinic services at the level of districts in individual regions of the Czech Republic in 2018. A three-phase analysis was performed, including an ArcGIS network analysis, multi-criteria evaluation according to the TOPSIS method, and a correlation analysis encompassing the confidence interval gained via the Bootstrap method. Seven indicators were selected—recipients of the allowance for the care, capacity of residential and outpatient-clinic services, and four indicators of accessibility via individual and public transport within the set time intervals. The results show good availability of residential care (no gap) within 30 min. by individual and public transport in most districts (94%). However, day services centers do not have a space gap in only 28% of districts by individual transport, and 8% of districts by public transport. In the case of day care centers, 54% of districts by individual transport, and 29% of districts by public transport do not have a space gap. The results also show that the level of spatial availability of care (gaps) in the district is not related to the number of people aged 65+ with reduced self-sufficiency in the district. On the contrary, the correlation analysis shows that with the growing number of people aged 65+ with reduced self-sufficiency in the district, the capacity of residential and outpatient services increases and the gaps in spatial accessibility do not decrease.

## 1 Introduction

Public policy within the area of social care focused on senior citizens (people over the age of 65) in the Czech Republic is part of the National Strategy of Social Services Development for the period between 2016 and 2025. This policy reacts again to the previously determined acute needs of society listed in the National Action Plan Supporting Positive Ageing for the period between 2013 and 2017 with an outlook to 2020. Besides other points, this deals with the provision of a wide offering of mutually connected social-health services for senior citizens and the provision of long-term and palliative care respecting the locality and time accessibility of services for senior citizens with limited self-sufficiency. The accessibility of social services is determined by the number of social services facilities present in the region and their capacity and transport reachability [1]. This fits into the broad stage of potential for care delivery talked about by [2]. In the Czech Republic, the limitedness of a person’s self-sufficiency is evaluated and examined by relevant bodies of the state administration. A person with limited self-sufficiency is then eligible for a financial allowance (benefit) from the state for social care. It is presumed that this person will use the allowance to purchase social services. [3,4].

The market of social services for the elderly has a relatively extensive network, which is provided in the form of outpatient, field, and residential services. Trends present in the developed countries of Europe show that residential social services for senior citizens are being replaced by field social care. An example is of this is the Norwegian market for social services for senior citizens where the ratio of home care rises along with the ratio of private social care providers [5]. A similar situation was observed in the markets for social services in Germany [6] and in France [7]. It turns out that a regular source of care and continuity of care significantly reduce the likelihood of hospitalizations in residential facilities [8]. The responsibility for the supply of social services in relation to their capacity, as well as spatial accessibility in the Czech Republic is held by self-governing regions. There are 13 regions and the capital city of Prague (further referred to as regions). By law, the regions create a network of social services with the necessary range and types of services (residential, field, outpatient-clinic services). Providers of social services are entities (organizations) from the public or private sectors. Some studies then point out the lower quality of private social services, [9–12]. Nevertheless, within the Czech Republic, residential social services prevail over field and outpatient-clinic types, and overall, the capacity of social services (supply) does not cover the desirability (demand) among citizens [13]. According to the results of an analysis performed by the MoLSA of the Czech Republic [14], an ideal state would be achieved by an increase in social services’ capacity by 36%.

This article is structured into five chapters. The first chapter is an introduction, which explains that the addressed problem is current and beneficial for all states that take care of the social needs of their citizens. The second chapter presents both an overview of professional literature and the key findings resulting from them, as well as the aim and hypothesis of the article. The third chapter is devoted to methodological content in addition to a description of the methods used, including characteristics of the data and selected parameters. The fourth chapter deals with the analysis and discussion of achieved results. The fifth chapter is a conclusion that briefly summarizes the findings and suggests opportunities for further research.

## 2 Theoretical background

The spatial accessibility and time accessibility of social services represent necessary preconditions of good quality of these services. Accessible services mitigate poverty and support independent life for citizens. Accessibility is a measure of supply and demand for social care resources, as well as the interaction between supply and demand within and between geographical regions [15]. Spatial accessibility is a topical issue in the whole of Europe and its optimal variants are the object of numerous studies [13,16]. Nursing infrastructure of social services for senior citizens in the Czech Republic is being studied mainly from the perspective of their capacity (number of beds in residential facilities, capacity of outpatient clinics), availability of human resources, and expenditures reserved in the budgets (state, regional, municipal), usually focusing only on residential social services for senior citizens [17,18]. Less attention is being paid to a complex evaluation of the quality of social services, except for the study on information accessibility of homes for the elderly [12] and the quality of services from the perspective of public and private providers [11]. Studies dealing with spatial accessibility of nursing infrastructure focusing on a complex supply of social services (outpatient-clinic, field, residential) are missing altogether.

Some basic preconditions for accessibility are a sufficient capacity of residential and outpatient-clinic care facilities and their spatial design including the specifics of the cities and countryside municipalities where they are located. A comparison of the accessibility and supply of social services in the cities and countryside in Estonia was conducted by [16]. The authors determined differences in the accessibility of some of the social services in relation to the size of the target group of clients and the nature of the services. Social services designated for wide groups of clients, so-called common social services (home care and consulting services), are more accessible in cities and municipalities, whereas social services designated for smaller groups of clients with specific needs (e.g. immobile persons), especially in the countryside, are less accessible. The authors came to the conclusion that the accessibility of social services for the most vulnerable people (senior citizens with lowered self-sufficiency) is a key attribute for the provision of social services as well as for the supportive care provided by close persons (relatives, neighbours). This fact is also confirmed by the results of analyses performed by [19,20,21] who state that the time spent commuting to social facilities is closely related to a person’s number of visits. It was revealed that longer distances to social facilities from the houses of relatives have a negative impact on the frequency of visits. According to [22], not only does distance play an important role, but also the difficulty of the transport connection. The diversity of the availability of services is perceived by [23] as whether the client goes to the service alone or with the help of an escort; whether he uses a private car, public transport, ambulance or walks, and how long it takes to arrive at the facility for service.

Spatial accessibility in relation to specific socio-economic issues is also currently being examined in Germany. [24] studied spatial transport accessibility of social services in rural areas and within the context of elderly citizens’ preferences. They used a quasi-experimental survey design focused on the citizens, couples over the age of 50 and persons over the age of 75, living in small countryside municipalities. The respondents were confronted with a hypothetical situation—the necessity of social care and the willingness to use residential care. The majority of respondents over the age of 50 prefer outpatient-clinic care at the place of their residence, and respondents over the age of 75 prefer residential care available at the place of their residence. The results of the Austrian study show that information on spatial accessibility of social facilities for the elderly, of course, plays an important role in supporting homes for the elderly and nursing homes. In this context, location and accessibility information seem to be particularly important for future clients and their family members [25].

From the perspective of clients and citizens, long-term care is generally preferred at the place of residence, at fist as outpatient-clinic (field) care and subsequently as residential long-term care. This is the general conclusion of research based on the examination of results from studies mostly conducted in European states and in the USA [26]. This is supported by results gained by [27] studying this within the conditions of Germany, and [28] focusing on Finland. The authors agree on the fact that regarding a population’s ageing, a decrease in informally established care (in families) for senior citizens is expected alongside an increased interest in formally established home and community care.

Author [29] evaluates nursing homes for senior citizens using spatial analysis and regression analysis in Chile. The author identifies clusters of nursing homes and estimates determinants of accessibility as well as coverage of the area by these services with regard to the incomes of individual municipalities. It was identified that richer municipalities provide much better care for senior citizens than poorer municipalities. Authors [30] studied the spatial accessibility of long-term-care services for senior citizens in the city of Wuhan, China. Attention was specifically focused on the identification of spatial differences in the accessibility of facilities for the care of senior citizens provided by private and public providers. This was performed using ArcGIS network analysis and the MCSG2SFCA method. It was confirmed that the spatial accessibility of all facilities for senior citizens is higher in urban areas, whereas the accessibility of care in border rural areas is considerably lower. Private facilities prevail mainly in developed urban areas. The development of private services of care for senior citizens depends on the density of the population in the central city and on the high standard of life. At the same time, the accessibility of the services in relation to bed capacity is relatively low, and the differences between the urban and rural areas are obvious. Similar results were obtained in the Australian research performed by [31]. The majority of residential facilities for senior citizens were established in the main cities whereas a lot of rural areas had a lower or just average supply of such services. According to [32], low accessibility of facilities for the care of senior citizens is caused not only by a low number of facilities, but also by their uneven spatial distribution.

In their qualitative study, [33] evaluate the time accessibility of social services for senior citizens in Beijing. The facilities for long-term care services for senior citizens are accessible in the majority of city parts within 30 minutes or less. For the suburban areas, time accessibility ranges from between 30 and 120 minutes. Time accessibility of the services of care for senior citizens is closely connected to the road network, transport services of urban and suburban areas, and the estimated standard speeds of travel. The results corroborate that senior citizens and their relatives mostly prefer social facilities located in a time distance lower than 60 minutes, and maximally 90 minutes, from their places of residence.

Authors [34] recommend that facilities of care for senior citizens flexibly react to demand from potential users of these services via establishing new facilities or increasing the capacity of existing ones, especially in distant areas where the accessibility of such services is low. In a case where a political decision is made in favour of increasing the capacity of existing facilities rather than increasing the number of these facilities, e.g. because of a budget limitation, the increase in capacity needs to be done in the closer facilities so that access for all senior citizens is facilitated.

The above findings are also developed by the authors of this article, whose ambition it is to verify the possibilities of a methodological approach based on a combination of multiple methods and to contribute with original outputs to better information about the actual state of spatial availability of social services in the Czech Republic.

Within this article, the object of the research is to represent the spatial accessibility of nursing services including residential and outpatient-clinic services in the Czech Republic for persons over the age of 65 with lowered self-sufficiency. The gap of spatial accessibility means the absence of the offer of the given services or their poor accessibility by individual and public transport in the district. The analysis of the spatial accessibility is structured into three logically interconnected phases. Each phase is built around the testing of a specific hypothesis (H1 –H3).

A number of measures have been proposed for the spatial dimensions of healthcare access within a given area, mainly availability and accessibility [15]. In the first phase of this paper, attention is focused on the determination of gaps in the spatial accessibility of residential and outpatient-clinic services. Spatial accessibility is understood as the transport reachability of the point where the care (service) is being provided, via the means of individual and public transport at the level of districts. This is based on a general presumption that individual and regional public transport (buses and trains) in the Czech Republic is provided via a sufficient and operational transport network [35,36]. Distance-based measures and spatial interaction models are some of the most commonly used methods for quantifying healthcare accessibility [15]. Distance-based measures are used to quantify traveling time or distance between patients and health care providers. Frequently, the measures used are distance (or time) to the nearest provider, the average distance (or time) to a set of providers, and the number of providers within a certain distance [2,37]. In this paper, the main measure is time-to-destination. ArcGIS network analysis at the level of districts of individual regions in the Czech Republic in 2018 is used.

H1: *“A good level of accessibility of residential and outpatient-clinic services*, *i*.*e*. *reachability within 30 min via the means of individual and public transport*, *is achieved by 80% of the districts in the Czech Republic*.*”*

In the second phase, the article deals with a multi-criteria evaluation of the spatial accessibility of residential and outpatient-clinic social services for the recipients of the allowance for care, according to the TOPSIS method, at the level of districts of individual regions in the Czech Republic in 2018. This method ranks the districts from the best to the worst.

H2: *“The best districts regarding spatial accessibility are the ones with the highest numbers of persons over the age of 65 with lowered self-sufficiency*, *and vice versa*.*”*

In the last part, the article focuses on the determination of the level of correlation between the number of recipients of the allowance for the care, the capacity of services, and the values representing the level of accessibility achieved via the TOPSIS method for 76 districts of the Czech Republic in 2018.

H3: *“With the increasing number of persons over the age of 65 with lowered self-sufficiency in a district*, *the capacity of residential and outpatient-clinic services rises*, *as does their spatial accessibility*.*”*

## 3 Data and methods

The infrastructure of social services consists of registered legal or natural persons who fulfil the requirements and the conditions defined in the law for the provision of social services in the Czech Republic, according to Act n. 108/2006 Coll., on Social Services, effective from 1 January 2007. The analysis of the spatial accessibility of providers of residential and outpatient-clinic services is focused on the area of individual districts. According to the data from [38], there were 6,258 municipalities and the capital city of Prague in the Czech Republic in 2018. From the perspective of the principle of composition, the municipalities determine the area of 76 districts which then determine the area of 13 regions (self-governing administrative bodies). A specific position within the self-governing structure is held by the capital city of Prague which is not divided into districts.

The capital city of Prague was not included in the analysis because of its incomparability with the districts or regions from the administrative, territorial, and demographic perspectives.

The data needed for the analyses were obtained from public databases, registers, sources provided by the relevant institutions, and original calculations. The main data sources included:

- Register of providers of social services in the Czech Republic, a source for getting and confirming the number of legitimate providers of social services and the addresses of places where social services are being provided so that the GPS coordinates could be obtained
- 13 regional networks of social services providers, source for the data on the capacity of individual types of social services
- Ministry of Labour and Social Affairs of the Czech Republic from which the numbers of persons over the age of 65 receiving the allowance for care according to individual stages of dependence by district in 2018 were obtained based on the request for the provision of information
- Information portal OKWork (OKPráce) and its information system together with the Integrated portal of the MoLSA of the Czech Republic, serving for the search of available locations within a defined time interval or within a defined distance when using the means of public transport. It describes the parameters of the connection between municipalities of the Czech Republic for commuting to work (ie. the chosen hours for the route there and back). The connection is normally searched for only among municipalities within a direct distance of less than 150 km. The database is then limited to the records where the commuting time is shorter than 90 minutes and the maximal number of changes is five. The search for connections was performed only at the level of municipalities (between the main stations in the start and target municipalities). The connections for this database were examined for arrivals at 7 AM, 8 AM and 2 PM. The connections were searched for in bus and train timetables, without local transport options.

### 3.1 Indicators

For the needs of the analysis of the spatial accessibility of social services for persons over the age of 65, seven indicators (X1 –X7) were studied. The indicators were selected so that they take into account not only the transport reachability of the social services providers and the capacity of the selected types of residential and outpatient-clinic social services in individual districts of the Czech Republic, but also the perspective of highly relevant demand represented by persons over the age of 65 (the elderly), being the recipients of the allowance for care from the state’s budget.

The first three indicators are X1, X2 and X3. Their values for the year of 2018 for 76 districts are listed in Table 1, aggregated according to the 13 regions of the Czech Republic.

**Table 1:** Indicators X1 –X3 for individual regions of the Czech Republic in 2018, * in thousands.

	CBR	SBR	PLR	CR	ÚR	LR	HKR	PR	VR	SMR	OR	MSR	ZR
X1*	25.4	15.4	13.5	6.0	18.9	10.3	13.9	13.5	13.6	30.6	16.0	30.4	16.8
X2*	4.9	3.4	2.5	1.3	4.8	1.4	2.5	2.4	2.3	5.9	3.3	5.2	3.0
X3	327	40	45	10	48	70	164	139	125	144	148	172	148

Source: [1].

Note: CBR—Central Bohemian Region, SBR—South Bohemian Region, PLR—Region of Pilsen, CR—Region of Karlovy Vary, ÚR—Region of Ústí, LR—Region of Liberec, HKR—Region of Hradec Králové, PR—Region of Pardubice, VR—Region of Vysočina, SMR—South Moravian Region, OR—Region of Olomouc, MSR—Moravian-Silesian Region, ZR—Region of Zlín.

**Indicator X1**—recipients of the allowance for care over the age of 65 (number of recipients). Social services are designated for people who are in unfavourable life situations and are at risk of being excluded from society. The largest group of social services users is represented by senior citizens (persons over the age of 65) who are dependent on help from another person because of lowered self-sufficiency or poor health conditions (dementia and other conditions). Persons with poor health over a long period of time who are not able to satisfy some of their basic life needs without help from others are eligible for a monthly social allowance—allowance for care. This allowance is provided based on Act n. 108/2006 Coll. on Social Services as amended, and Regulation n. 505/2006 Coll. for the application of some of the clauses from the Act on Social Services as amended. The amount of the allowance depends on the age of the claimant (until 18 years of age, above 18 years of age) and the stage of dependence on help from another person (stage I.–light dependence, stage II.–moderately serious dependence, stage III.–serious dependence, stage IV.–full dependence). The allowance for care serves as the payment for social services provided. In general, social services, especially residential ones, are primarily designated for persons with a declared stage of dependence, i.e. the recipients of the allowance for care.

**Indicator X2**—capacity of the providers of residential social services (capacity of residential). Residential social services include homes for the elderly and special regime homes. These are social facilities in which long-term social care is provided, especially for persons over the age of 65 who are dependent on help from another person because of their lowered self-sufficiency and poor health conditions (dementia or other conditions) related to their advanced age.

**Indicator X3**—capacity of outpatient-clinic social services (capacity of clinic). Outpatient-clinic social services include day care centres and centres providing day services. These social services are provided in facilities which are only visited by clients, i.e. overnight accommodation, short-term accommodation, or long-term accommodation are not provided. Day care centres are designated for persons with lowered self-sufficiency for whom full-day, complex care is provided with regard to the needs of individual clients, including educational and activating activities. Day services centres provide help with personal hygiene and with the arrangement of the personal affairs of a client.

There were 760 residential and 582 outpatient-clinic social services facilities for persons over the age of 65 identified in the Czech Republic (excluding the capital city of Prague) in 2018. Th highest proportion was held by homes for the elderly (493) and special regime homes (114) with a total capacity of above 40 thousand beds. In the case of the outpatient-clinic social services, there was everyday care available for senior citizens in day care centres (100) and centres providing day services (33) with the total capacity of 1,580. The average daily capacity for outpatient-clinic social services ranges between 5 to 15 spots.

Then, there are the four indicators (X4 –X7) representing spatial (transport) reachability of residential and outpatient-clinic social facilities via the means of individual and public transport (buses and trains). These are calculated in regard to the places of residence of the recipients of the allowance for care over the age of 65 in individual districts. The values of these indicators are the results of original calculations and they represent the level of coverage of the districts’ areas by the services given, from the perspective of the recipients of the allowance (X1) living within the time area given.

**Fig 1:**
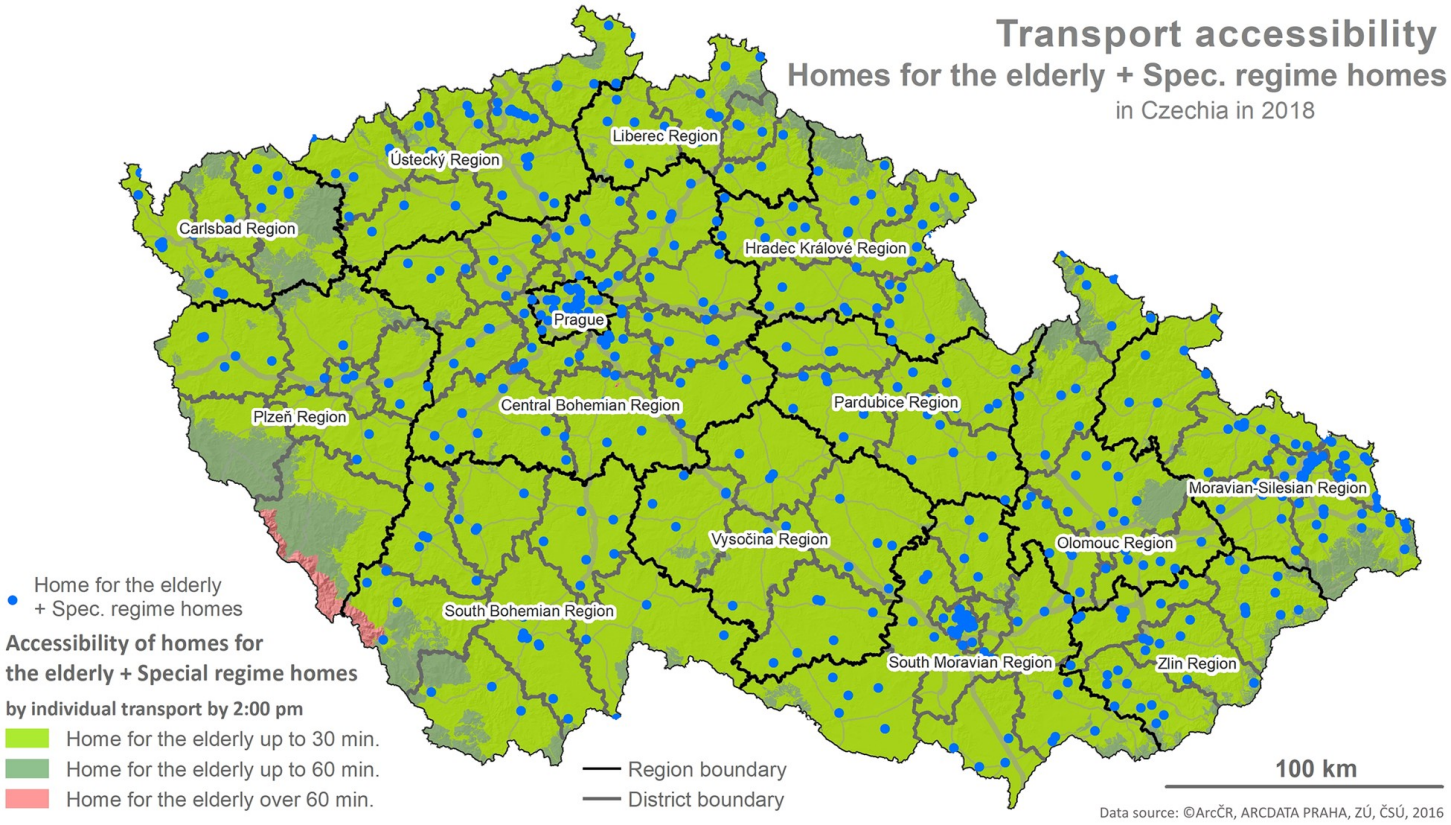
Accessibility via the means of individual transport. Printed using ArcCR ^®^500 Geographical Data Model version 3.3 under a CC BY 4.0 license, original copyright 2016.

**Fig 2:**
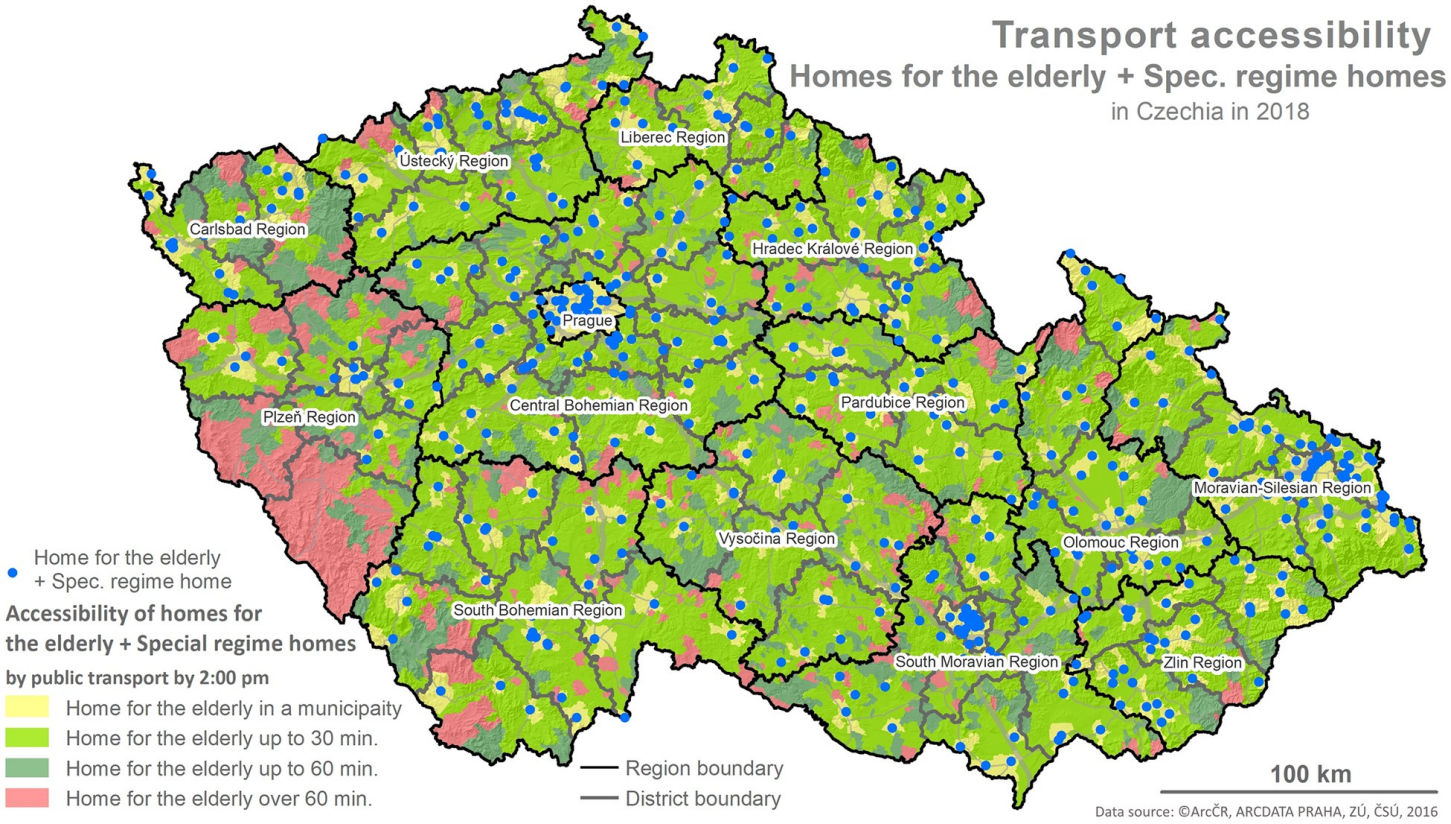
Accessibility via the means of public transport. Printed using ArcCR ^®^500 Geographical Data Model version 3.3 under a CC BY 4.0 license, original copyright 2016.

**Fig 3:**
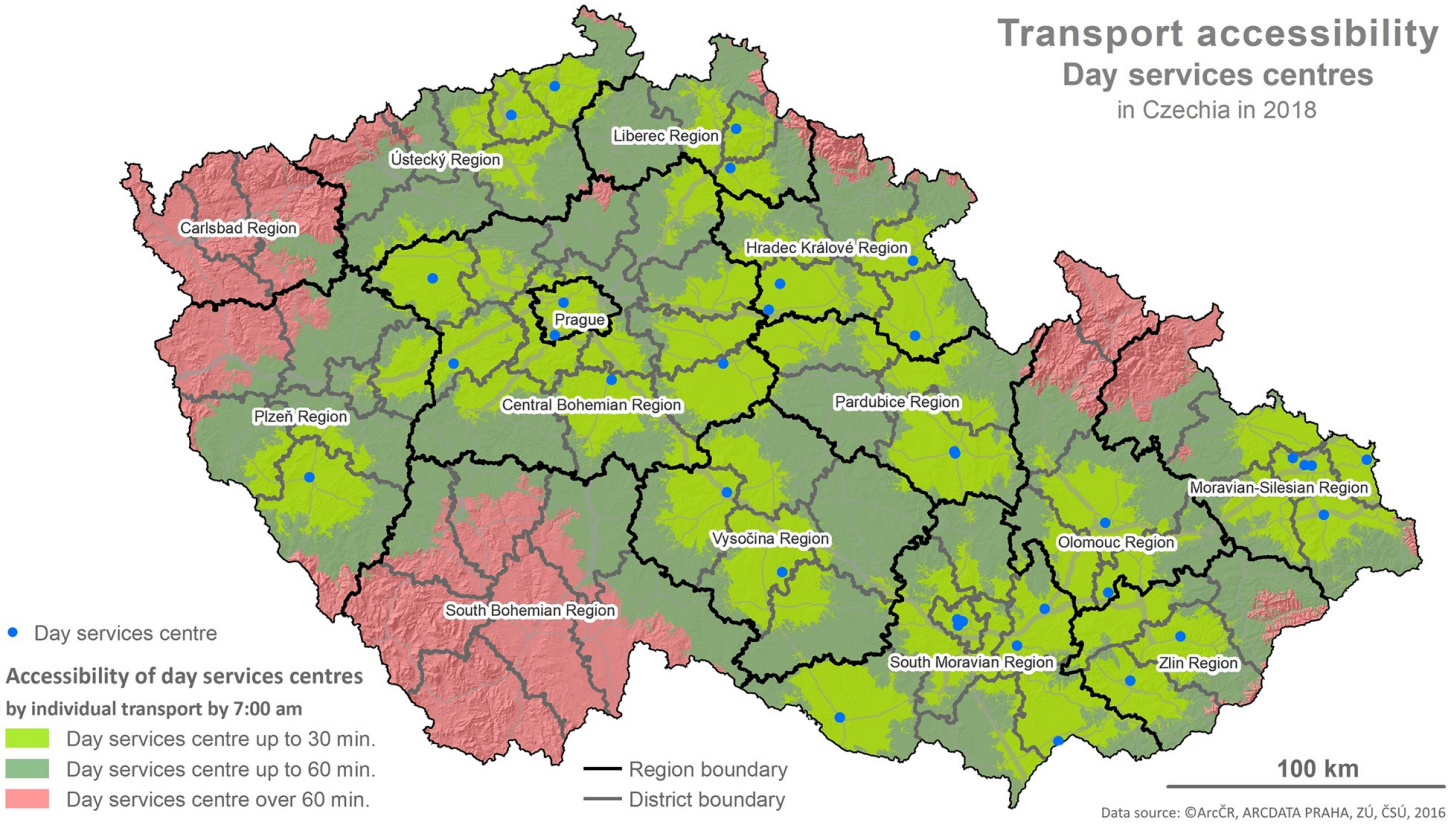
Accessibility via the means of individual transport. Printed using ArcCR ^®^500 Geographical Data Model version 3.3 under a CC BY 4.0 license, original copyright 2016.

**Fig 4:**
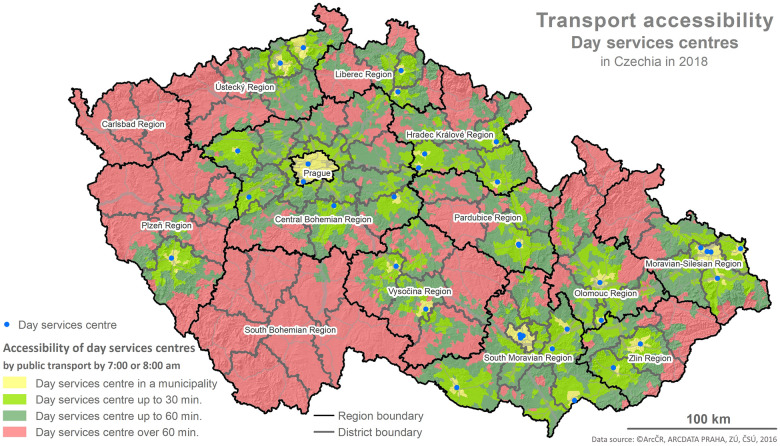
Accessibility via the means of public transport. Printed using ArcCR ^®^500 Geographical Data Model version 3.3 under a CC BY 4.0 license, original copyright 2016.

**Fig 5:**
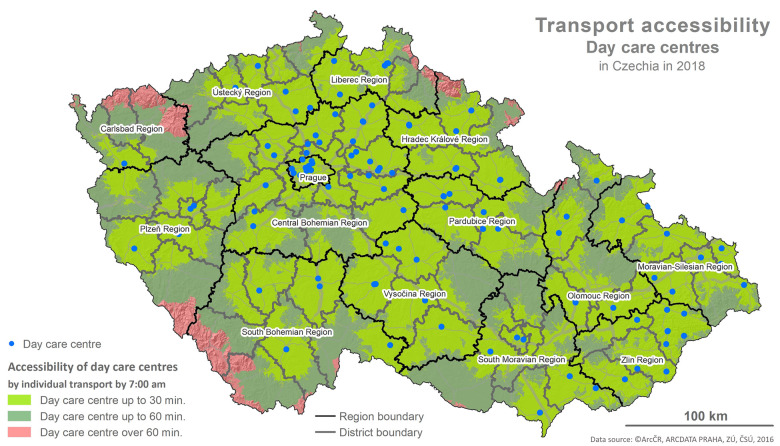
Accessibility via the means of individual transport. Printed using ArcCR ^®^500 Geographical Data Model version 3.3 under a CC BY 4.0 license, original copyright 2016.

**Fig 6:**
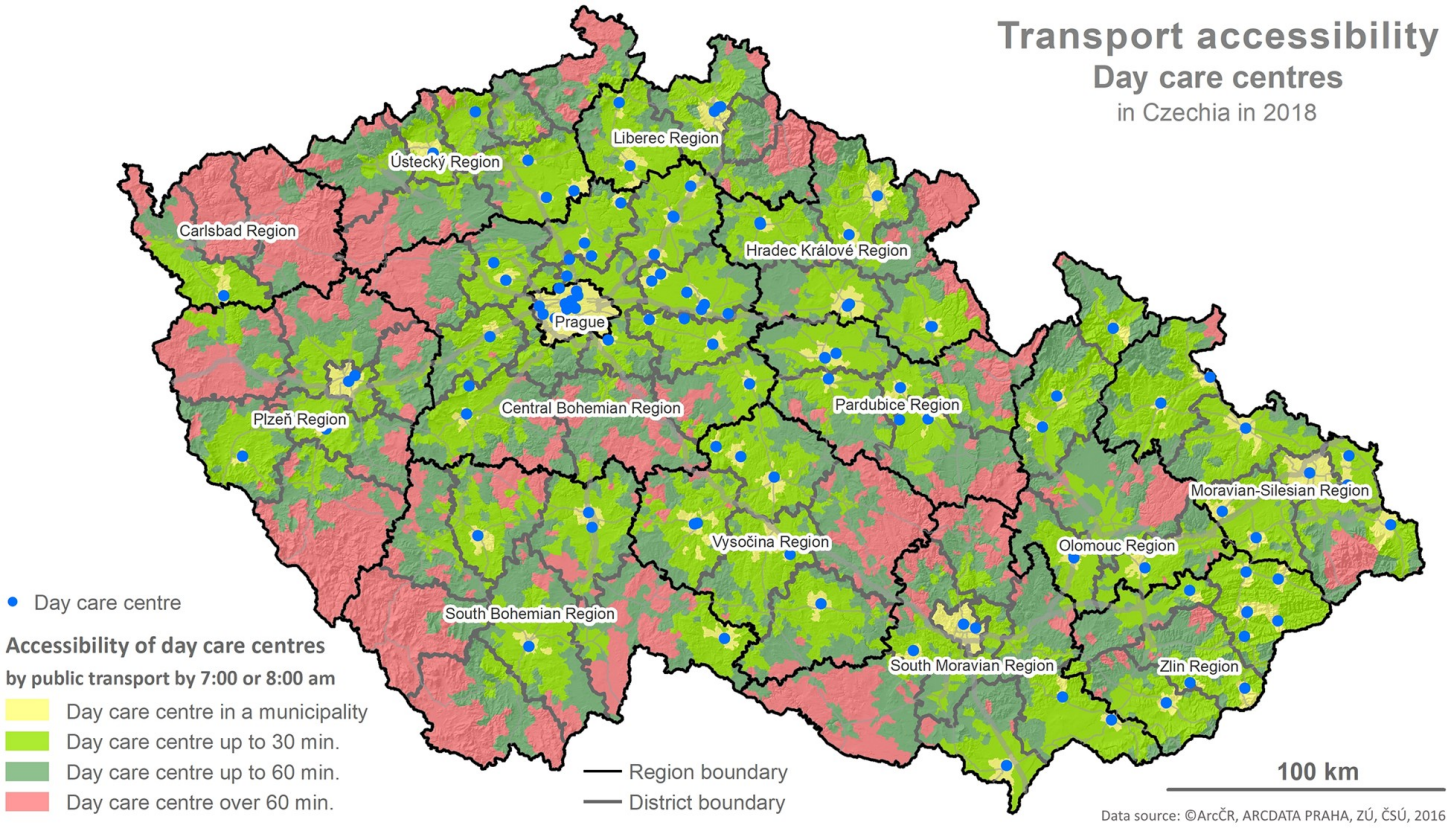
Accessibility via the means of public transport. Printed using ArcCR ^®^500 Geographical Data Model version 3.3 under a CC BY 4.0 license, original copyright 2016.

The foundation for the calculations is represented by the values of the indicator X1 and the determination of reachability using the network analysis following the methodology described below and depicted in the maps (Figs 1–6).

**Indicator X4** is decomposed into X4_a_ and X4_b_, and it represents the transport reachability of residential services (R, homes for the elderly + spec. regime homes) via the means of individual transport (IT) in intervals within 30 min and 60 min at 2 PM on Tuesdays for individual districts (*d*_*n*_) for persons (X1) living in the time area given, in %, according to formula (1):

$$X4=\;(\Sigma X1_{dn,\;IT\;R\;to\;30\;min.,\;to\;60\;min.}/\Sigma X1_{dn}).100$$ (1)

**Indicator X5** is decomposed into X5_a_ and X5_b_, and it represents the transport reachability of residential services (R, homes for the elderly + spec. regime homes) via the means of public transport (PT) in intervals within 30 min and 60 min at 2 PM on Tuesdays for individual districts (*d*_*n*_) for persons (X1) living in the time area given, in %, according to formula (2):

$$X5=\;(\Sigma X1_{dn,\;PT\;R\;to\;30\;min.,\;to\;60\;min.}/\Sigma X1_{dn}).100$$ (2)

**Indicator X6** is decomposed into X6_a_ and X6_b_, and it represents the transport reachability of outpatient-clinic services (C, day services centres + day care centres) via the means of individual transport (IT) in intervals within 30 min and 60 min at 7 AM and 8 AM on Tuesdays for individual districts (*d*_*n*_) for persons (X1) living in the time area given, in %, according to formula (3):

$$X6=\;(\Sigma X1_{dn,\;IT\;C\;to\;30\;min.,\;to\;60\;min.}/\Sigma X1_{dn}).100$$ (3)

**Indicator X7** is decomposed into X7_a_ and X7_b_, and it represents the transport reachability of outpatient-clinic services (C, day service centres+ day care centres) via the means of public transport (PT) in intervals within 30 min and 60 min at 7 AM and 8 AM on Tuesdays for individual districts (*d*_*n*_) for persons (X1) living in the time area given, in %, according to formula (4):

$$X7=\;(\Sigma X1_{dn,\;PT\;C\;to\;30\;min.,\;to\;60\;min.}/\Sigma X1_{dn}).100$$ (4)

### 3.2 Methodology of the evaluation of the spatial accessibility of social services

The foundation for the analysis of the spatial accessibility of social services is created from the coordinates of the locations of individual providers. Two time intervals for commuting were set, within 30 min and within 60 min, and a specific day of the week was selected, a so-called common workday. Tuesday was selected as a common workday since Mondays and Fridays are often affected by weekend commuting. For the case of public transport, only connections with an arrival time of 7 AM, 8 AM or 2 PM on a common workday were selected. This was the same for individual transport. The result of the selection analysis is represented by the records on the connections meeting the conditions defined in the methodology. From these records, the most suitable connections were selected using a weighing function, comparing the time of travel, time of arrival, time of departure, number of changes, and price. The facilities of the selected social service are located at various places within the municipalities. It needs to be emphasized that city public transport was excluded from the analysis. Therefore, the actual results of the analysis do not describe the accessibility of the facilities (i.e. the closest bus stops near these facilities) but rather the accessibility of the municipalities (main stations, bus stops) in which the facilities are located. The access zones of these municipalities are determined by the time of travel via the means of public transport (including the time needed for changing and waiting for another connection).

A geographical information system (GIS) represents a framework for the collection, management, analysis, and visualisation of data. A GIS enables the analysis of spatial location, organization of data in layers, and data visualisation in the form of maps and 3D scenes [39]. Organization of data in layers has rules in which different types of data are stored in two basic data model types—vectors and rasters. For the case of the vector data, it is then distinguished whether the object will be represented as a point, line, or polygon [40]. The most significant element of a GIS is its ability to perform spatial analyses where the basic types of analyses include network analysis [41]. These analyses are mainly used when evaluating transport accessibility [42]. For this work, tools for the modelling of access zones (service areas) implemented in the ArcGIS environment were used [39]. The access zones in the map are visualized as polygons with clearly defined borders.

To model the transport accessibility via the means of individual transport, a network analysis technique was used. Network analyses in a GIS rely on algorithms whose theoretical cores are based on graph theory [43]. The usual types of tasks being solved this way include: the search for the best route (Best Route), the search for the closest facility (Closest Facility), the search for the best route among a set of resources and a set of targets (OD), Cost Matrix, modelling of the access zones, and other accessibility analyses [44]. The access zones are areas defined in relation to a network (i.e. road network) that include all sections of the network (roads) accessible from the centre being studied, under the set conditions. The result of the analysis consists of concentric areas created by the time intervals being studied.

Generation of access zones for the selected facilities was performed using a geoprocessing service denoted as “service area” that is published on a cloud GIS solution called ArcGIS Online. An advantage of this service is its ability to include information on traffic (current, historical, or predictive), [45]. This makes the estimations of the access zones via the means of individual transport more realistic.

Much has been written in social or health studies about the spatial accessibility of long-term care services, especially through the use of spatial models in a geographic information system (GIS), for example in the USA [2], in South Africa [46], in Japan [47] and in Toronto [48].

The accessibility of the facilities of the selected social services via the means of public transport was defined using the database of transport connections [49,50]. The database is regularly updated and published as a CSV file for the Czech Republic. Based on the search in the specialized literature, and consultations with the users of outpatient-clinic and residential services, including relatives, two time intervals were determined (see Table 2). These intervals were defined as main criteria for the evaluation of the accessibility of the selected social services via the means of individual and public transport.

**Table 2:** Criteria and weights for time intervals of the accessibility of social services being evaluated using TOPSIS.

Social services for persons 65+	Time interval	Individual transport			Public transport		
		Weight	Indicators		Weight	Indicators	
Residential	within 30 min	0.60	X4_a_	X4	0.60	X5_a_	X5
	within 60 min	0.40	X4_b_		0.40	X5_b_	
Clinic	within 30 min	0.70	X6_a_	X6	0.70	X7_a_	X7
	within 60 min	0.30	X6_b_		0.30	X7_b_	

Source: Original elaboration.

### 3.3 Multi-criteria evaluation using the TOPSIS method

The TOPSIS method (Technique for Order of Preference by Similarity to Ideal Solution) belongs among multi-criteria decision-making methods that were developed over a long period of time by authors Hwang and Yoon [51] as an alternative to the ELECTRE method [52]. The purpose of the TOPSIS method is to select the alternatives that are closest to the ideal solution and furthest from the base alternative. The result of the TOPSIS technique is described by [53] and [54] as a solution with the shortest distance from the ideal solution calculated using Euclidean distance.

The procedure of the calculation using the TOPSIS method can be described via the following steps:

1. creation of a matrix D in which the alternatives are ordered according to the relevant, pre-defined criteria,
  $$D=(\begin{array}{ccccc} & X_{1} & X_{2}\ldots & X_{j}\ldots & X_{n}\\ A_{1} & x_{11} & x_{12}\ldots & x_{1j}\ldots & x_{1n}\\ A_{2} & x_{21} & x_{22}\ldots & x_{2j}\ldots & x_{2n}\\ : & : & : & : & :\\ A_{i} & x_{i1} & x_{i2}\ldots & x_{ij}\ldots & x_{in}\\ : & : & : & : & :\\ A_{m} & x_{m1} & x_{m2}\ldots & x_{mj}\ldots & x_{mn} \end{array})$$
  where *A*_*i*_ = *ith* alternative, *x*_*ij*_ = value of *jth* criterion reached by *ith* alternative.
2. creation of a normalized criteria matrix that serves for the calculation of normalized values, following formula (5):
  $$r_{ij}=x_{ij}/\sqrt{\overset{j}{\underset{j=1}{\sum }}x_{ij}^{2}}$$ (5)
  where *x*_*ij*_ = value of *jth* criterion reached by *ith* alternative.
3. multiplication by the weights for each individual criterion, following formula (6):
  $$v_{ij}=w_{ij}\cdot r_{ij}$$ (6)
  where *v*_*ij*_ = weighted normalized value and *w*_*ij*_ = criterion’s weight.
4. determination of the ideal alternative *H*_*j*_ and the base alternative *D*_*j*_, following formula (7):
  $$H_{j}=\text{max}(w_{ij}),\;D_{j}=\;\text{min}(w_{ij})$$ (7)
5. calculation of the alternatives’ distances from the ideal alternative and the base alternative, following formula (8):
  $$d_{i}^{+}={\left[\overset{k}{\underset{j=1}{\sum }}{\left(w_{ij\;}-H_{j}\right)}^{2},\right]}^{1/2},\;d_{i}^{-}=\;{\left[\overset{k}{\underset{j=1}{\sum }}{\left(w_{ij}-D_{j}\right)}^{2},\right]}^{1/2}$$ (8)
6. calculation of the relative distance from the base alternative, following formula (9):
  $$c_{i}=\;\frac{d_{i}^{-}}{d_{i}^{-}+\;d_{i}^{+}}$$ (9)
  where c_i_ is the indicator of the relative distance from the base alternative.

The result values of the *c*_*i*_ indicator are within the interval <0, 1> where 0 is represented by the base alternative, and 1 is represented by the ideal alternative. The alternatives are then ordered according to the *c*_*i*_ indicator’s values from highest to lowest based on which complete order of alternatives is being evaluated [55].

An important step in the calculation using the TOPSIS technique is the determination of the weights for the criteria that reflect the relative importance of each individual criterion. Weights can be determined, for example, using the method of order, the method of points, Saaty’s method, or Fuller’s triangle [55]. According to [56], weights for the criteria can be determined subjectively, expertly, objectively, or in an integrated manner (a combination of the first and second approaches).

The TOPSIS method is also used to evaluate the performance, quality of services, and programs in the public and private sectors. In particular, the publications of authors [57–59] who have focused their research on supply chain and transport selection. As the authors state, the TOPSIS method represents a suitable and supportive tool for the process of selection or evaluation of a defined segment, from the point of view of defined evaluation criteria (quantitative and qualitative). This process is one of the most important factors and has a direct impact on the performance of the organization and the entire process.

Weights for the eight criteria being studied (indicators X4_a, b_−X7_a, b_) were assigned using an integrated approach (see Table 2). Expert consultations and interviews with the clients of social services and their relatives were conducted during February and March 2020. The weights for the criteria of accessibility of social services for senior citizens were determined using a scale of 0–1, where 0 represents the lowest weight and 1 represents the highest weight.

### 3.4 Correlation analysis and the Bootstrap method

The relationship between two attributes, including intervals, is only measured using Pearson’s coefficient of linear correlation (denoted as *r*). This is calculated using the covariance, i.e. the common dispersion of two variables where each deviation from the average of one variable $\left(x_{i}-\overset{-}{x}\right)$ is multiplied by the deviation from the average of the second variable $\left(y_{i}-\overset{-}{y}\right)$, and these deviations are subsequently added together and divided by N − 1, following formula (10):

$$cov(x,\;y)=\frac{\sum (x_{i}-\bar{x})(y_{i}-\bar{y})}{N-1}$$ (10)

This covariance is standardized following formula (11):

$$r=\frac{cov_{xy}}{S_{x}S_{y}}$$ (11)

Pearson’s coefficient is a symmetric coefficient, with values within 〈−1, +1〉, and the fact that it is a coefficient of linear correlation means that it can only capture the relationship between two variables. A linear relationship occurs when, with the changing values of one variable, the values of the other variable change proportionally.

Pearson’s coefficient was tested for two significance levels, *α* = 0.05 and *α* = 0.01. An alternative to the tests of statistical significance is represented by the confidence interval (CI) for the correlation coefficient *r*. For this purpose, the bootstrap method was applied.

Bootstrapping is a computer technique that simulates a population based on an original sample, from which the computer generates multiple further samples (so-called bootstrap samples) with the same size as that of the original sample. The lower and the upper border of the CI are determined using the percentile method for 95%.

Pearson’s coefficient and the confidence interval using the bootstrap method were calculated in IBM SPSS Statistics software [60].

## 4 Results and discussion

The results of the programming and calculations performed are described in steps. At first, attention is focused on the evaluation of the visual (map) form of the spatial accessibility of individual types of social services in the ArcGIS environment. This is followed by the multi-criteria evaluation of the spatial accessibility using the TOPSIS method and the evaluation of the relationship between the selected indicators using Pearson’s coefficient and the bootstrap method.

### 4.1 Spatial accessibility

The evaluation of the spatial accessibility via the means of individual and public transport for social services (residential and outpatient-clinics) for the times set (see Table 2) was performed using the map outputs (Figs 1–6) and using an original three-tier scale (I., II., III.) for the level of transport accessibility. The scale of the accessibility takes into account the time to reach the place providing the social service from the client’s place of residence, the transport coverage of the district’s area in % by the service given, and the presence of the service itself in the district given.

The scale of the spatial accessibility:

1. good accessibility (no gaps): there is at least one provider of the service given with a seat available in the district and the reachability, for the most part, of the district’s area (80–100%) is within 30 min;
2. lowered accessibility (with a gap): regardless of whether the provider’s available seat is in the district’s area, a reachability within 30 min is only secured for 50–70% of the district’s area, and the rest of the district’s area has a reachability within 60 min or above 60 min;
3. poor accessibility (with a significant gap): there is no provider of the service given with a seat available in the district, and the reachability is above 60 min for most of the area (60%).

#### 4.1.1 The results for the spatial accessibility of residential services: Homes for the elderly and special regime homes

The spatial accessibility via the means of individual and public transport to the locations of residential services (homes for the elderly and special regime homes in individual districts) is displayed in two separate maps depicting the area of the Czech Republic, regions and the districts within them (see Figs 1 and 2). The colours distinguish the time reachability within each of the time intervals set (see the legend). Blue dots represent the locations of residential service facilities.

Even the visual comparison suffices for identification of the fact that the spatial accessibility is slightly better in the case of individual transport than it is for public transport, especially in the Regions of Pilsen, Karlovy Vary and South Bohemia. Among these three regions, the situation is worst in the Region of Pilsen, specifically in the Klatovy and Domažlice Districts where there are no available seats in residential services. The area of these districts is partially covered by the Šumava Mountains, same as in the Prachatice District, creating a natural transport obstacle. In the case of the Karlovy Vary District in the Region of Karlovy Vary, reachability is affected by the nature of the territory where the Hradiště Military Area is in the east.

An aggregate view of the reachability is shown in Table 3 which includes the scale-based evaluation (I.–good, II. lowered, III.–poor) of the spatial accessibility. The very left column lists the regions of the Czech Republic (excluding the capital city of Prague) and the brackets contain the total number of districts within the region given. The other columns list the number of districts meeting each level of the scale for the spatial accessibility via the means of individual and public transport. The last row of the table shows the portion of the districts in % within the scale for all the regions.

**Table 3:** Homes for the elderly and special regime homes: Accessibility scale by the regions.

Regions (number of districts)	Accessibility via the mans of individual transport			Accessibility via the means of public transport		
	I.	II.	III.	I.	II.	III.
CBR (12)	12	0	0	12	0	0
SBR (7)	7	0	0	5	2	0
PLR (7)	5	0	2	3	2	2
CR (3)	2	1	0	1	2	0
ÚR (7)	7	0	0	7	0	0
LR (4)	4	0	0	4	0	0
HKR (5)	5	0	0	5	0	0
PR (4)	4	0	0	4	0	0
VR (5)	5	0	0	5	0	0
SMR (7)	7	0	0	7	0	0
OR (5)	5	0	0	5	0	0
MSR (6)	6	0	0	6	0	0
ZR (4)	4	0	0	4	0	0
Total in %	96,1	1,3	2,6	89,5	7,9	2,6

Source: Own elaboration.

Following the results of the scale-based analysis, it can be stated that the spatial accessibility of homes for the elderly and special regime homes from the perspective of the transport utilized (individual, public) is comparable, and for more than 89% of districts (96,1% in the case of individual transport and 89,5% in the case of public transport) it applies that: there is at least one provider of the service given with an available seat in each district and the reachability for the most part of the district’s area (80–100%) is within 30 min.

#### 4.1.2 The results of the spatial accessibility of outpatient-clinic services: Day services centres and day care centres

The spatial accessibility via the means of individual and public transport for the case of seats available in outpatient-clinic services (day services centres, day care centres) is displayed in four separate maps depicting the area of the Czech Republic, regions and the districts within them (see Figs 3–6). Same as in the case of residential services, the colours in the maps distinguish the time reachability for the selected time intervals (see the legend). Blue dots represent available seats in the outpatient-clinic services.

The maps in Figs 3 and 4 depict the spatial accessibility of the outpatient-clinic services in the form of day services centres. The visual comparison shows that spatial accessibility is considerably better for the means of individual transport than for public transport. To a great extent, the poor accessibility is caused by the generally low number of facilities providing to these services in the Czech Republic, followed by the absence of these services not only in many districts but also in two whole regions (The South Bohemian Region, The Region of Karlovy Vary).

An aggregate view of the reachability is shown in Table 4 which includes the scale-based evaluation (I.–good, II. lowered, III.–poor) of the spatial accessibility. The structure of the data listed in Table 4 follows the same logic as in Table 3, where further description was included.

**Table 4:** Day services centres: Accessibility scale by the regions.

Regions (number of districts)	Accessibility via the means of individual transport			Accessibility via the means of public transport		
	I.	II.	III.	I.	II.	III.
CBR (12)	3	9	0	1	9	2
SBR (7)	0	0	7	0	0	7
PLR (7)	0	6	1	0	1	6
CR (3)	0	0	3	0	0	3
ÚR (7)	1	5	1	1	4	2
LR (4)	1	3	0	1	2	1
HKR (5)	2	3	0	0	4	1
PR (4)	1	3	0	0	2	2
VR (5)	1	4	0	0	2	3
SMR (7)	5	2	0	1	6	0
OR (5)	2	1	2	0	3	2
MSR (6)	3	2	1	2	3	1
ZR (4)	2	1	0	0	3	1
Total in %	28	52	20	8	51	41

Source: Own elaboration.

Following the results of the scale-based analysis, it can be stated that the spatial accessibility of day services centres from the perspective of the transport utilized (individual, public) is not fully comparable. Excluding the area of the available seat at the facility providing the service given, some similarity is indicated by lowered spatial accessibility (see stage II. of the scale used) where 51–52% of the districts have a lowered level of spatial accessibility via the means of individual as well as public transport. For these districts, it applies that regardless of whether the provider’s seat is in the district’s area, a reachability within 30 min is only secured for 50–70% of the district, and the rest of the district’s area has a reachability within 60 min or above 60 min. Nonetheless, a poor level of accessibility was detected for 20% of the districts (13) when regarding individual transport and for 41% of the districts (31) when regarding public transport. For these districts, it applies that there is no provider of the service given with an available seat in the district and the reachability is above 60 min for the most part of the area (60%). A good level of accessibility was detected for 28% of the districts (21) using the means of individual transport and only for 8% of the districts (6) using the means of public transport.

The maps in Figs 5 and 6 depict the spatial accessibility for outpatient-clinic types of services in the form of day care centres. A visual comparison reveals that spatial accessibility is better by means of individual transport than by means of public transport. To a great extent, the poor level of accessibility is caused by the generally low number of facilities providing these services in the Czech Republic, followed by the absence of these services in numerous districts.

An aggregate view of the reachability is displayed in Table 5 which includes the scale-based evaluation (I.–good, II. lowered, III.–poor) of the spatial accessibility. The data listed in Table 5 follow the same logic as presented in Tables 3 and 4 above.

**Table 5:** Day care centres: Accessibility scale by the regions.

Regions (number of districts)	Accessibility via the means of individual transport			Accessibility via the means of public transport		
	I.	II.	III.	I.	II.	III.
CBR (12)	9	3	0	4	6	2
SBR (7)	3	4	0	1	4	2
PLR (7)	3	4	0	1	4	2
CR (3)	0	3	0	0	1	2
ÚR (7)	3	4	0	3	3	1
LR (4)	2	2	0	1	3	0
HKR (5)	3	2	0	1	4	0
PR (4)	2	2	0	1	3	0
VR (5)	4	1	0	2	2	1
SMR (7)	3	4	0	1	5	1
OR (5)	1	4	0	2	3	0
MSR (6)	4	2	0	4	2	0
ZR (4)	4	0	0	1	3	0
Total in %	54	46	0	29	57	14

Source: Own elaboration.

Following the scale-based analysis, it can be stated that the spatial accessibility of day care centres from the perspective of the transport utilized (individual, public) is comparable to only a very small extent. A good level of accessibility (see stage I. of the scale used) for day care centres was detected for 54% of the districts (41) via the means of individual transport and only for 29% of the districts (17) via the means of public transport. For these districts, it applies that: there is at least one provider of the service given with an available seat in the district and the reachability for the most part of the district’s area (80–100%) is within 30 min.

A lower level of spatial accessibility (see stage II. of the scale used) of day care centres was detected for 46% of the districts (35) by means of individual transport and for 57% of the districts (53) by means of public transport. For these districts it applies that: regardless of whether the provider’s available seat is in the district’s area, a reachability within 30 min is only secured for 50–70% of the district’s area, and the rest of the district’s area has a reachability within 60 min or above 60 min.

A poor level of spatial accessibility (see stage III. of the scale used) was not detected for any of the districts by means of individual transport and it was detected for 14% of the districts (11) by means of public transport. For these districts, it applies that: there is no provider of the service given with an available seat in the district and the reachability is above 60 min for the most part of the area (60%).

### 4.2 The results of the spatial accessibility according to the TOPSIS analysis

**Fig 7:**
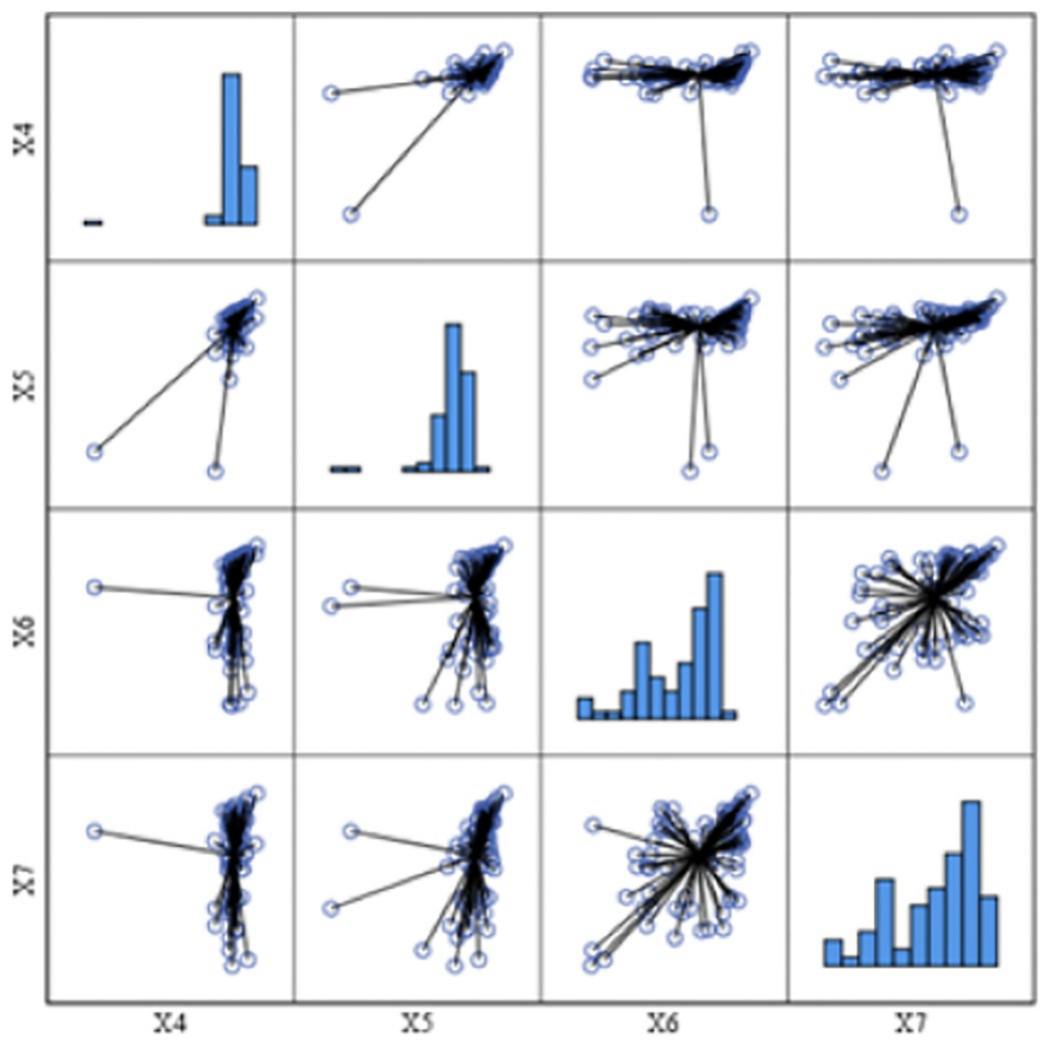
Distribution matrix of *c*_*i*_ for the indicators X4 –X7.

The results of the multi-criteria analysis are interpreted using the distribution matrix of *c*_*i*_ for the corresponding four indicators: X4*c*_*i*_ (residential service, IT), X5*c*_*i*_ (residential service, PT), X6*c*_*i*_ (clinic service, IT) a X7*c*_*i*_ (clinic service, PT), see Fig 7.

A static description of X4: mean value of *c*_*i*_ = 0.8631, median of *c*_*i*_ = 0.8720, standard deviation of *c*_*i*_ = 0.1041, minimum of *c*_*i*_ = 0.059, maximum of *c*_*i*_ = 1.00. Deviation (D20)–the Domažlice District (PR). Skewness (as an absolute value) of *c*_*i*_ = 6.247, kurtosis of *c*_*i*_ = 48.449.

From the ranking, the first five (best) districts are: D56 Brno—City *c*_*i*_ = 0.999, D57 Brno—Countryside *c*_*i*_ = 0.992, D28 Karlovy Vary *c*_*i*_ = 0.948, D35 Teplice *c*_*i*_ = 0.944, D41 Hradec Králové *c*_*i*_ = 0.938. The last five (worst) districts are: D20 Domažlice *c*_*i*_ = 0.005, D71 Bruntál *c*_*i*_ = 0.753, D54 Žďár nad Sázavou *c*_*i*_ = 0.758, D21 Klatovy *c*_*i*_ = 0.758, D73 Karviná *c*_*i*_ = 0.805.

A static description of X5: mean value of *c*_*i*_ = 0.8358, median of *c*_*i*_ = 0.8820, standard deviation of *c*_*i*_ = 0.1503, minimum of *c*_*i*_ = 0.001, maximum of *c*_*i*_ = 1.00. Deviations (17, 20, 21)–the Prachatice District (SBR), the Domažlice District and the Klatovy District (PR). Skewness of *c*_*i*_ = 3.945, kurtosis of *c*_*i*_ = 18.680.

From the ranking, the first five (best) districts are: D56 Brno—City *c*_*i*_ = 1.000, D23 Plzeň –City *c*_*i*_ = 0.958, D35 Teplice *c*_*i*_ = 0.948, D38 Jablonec nad Nisou *c*_*i*_ = 0.944, D36 Ústí nad Labem *c*_*i*_ = 0.942. The last five (worst) districts are: D21 Klatovy *c*_*i*_ = 0.001, D20 Domažlice *c*_*i*_ = 0.114, D17 Prachatice *c*_*i*_ = 0.532, D24 Plzeň –North *c*_*i*_ = 0.673, D54 Žďár nad Sázavou *c*_*i*_ = 0.688.

A static description of X6: mean value of *c*_*i*_ = 0.7011, median of *c*_*i*_ = 0.7985, standard deviation of *c*_*i*_ = 0.2471, minimum of *c*_*i*_ = 0.0780, maximum of c_i_ = 1.00. Skewness (as an absolute value) of c_i_ = 0.862, kurtosis of c_i_ = 0.244.

From the ranking, the first five (best) districts are: D56 Brno—City c_i_ = 1.000, D35 Teplice c_i_ = 0.963, D41 Hradec Králové c_i_ = 0.963, D57 Brno—Countryside c_i_ = 0.954, D23 Plzeň –City c_i_ = 0.952. The last five (worst) districts are: D29 Sokolov c_i_ = 0.078, D17 Prachatice c_i_ = 0.084, D13 České Budějovice c_i_ = 0.094, D28 Karlovy Vary c_i_ = 0.153, D14 Český Krumlov c_i_ = 0.281.

A static description of X7: mean value of c_i_ = 0.6400, median of c_i_ = 0.7175, standard deviation of *c*_*i*_ = 0.2510, minimum of *c*_*i*_ = 0.00, maximum of c_i_ = 1.00. Skewness (as an absolute value) c_i_ = 0.812, kurtosis *c*_*i*_ = 0.344.

From the ranking, the first five (best) districts are: D56 Brno—City c_i_ = 0.997, D23 Plzeň –City c_i_ = 0.966, D35 Teplice c_i_ = 0.944, D76 Ostrava—City c_i_ = 0.931, D4 Kolín c_i_ = 0.912. The last five (worst) districts are: D29 Sokolov c_i_ = 0.000, D28 Karlovy Vary c_i_ = 0.035, D17 Prachatice c_i_ = 0.090, D61 Znojmo c_i_ = 0.162, D30 Děčín c_i_ = 0.202.

**Fig 8:**
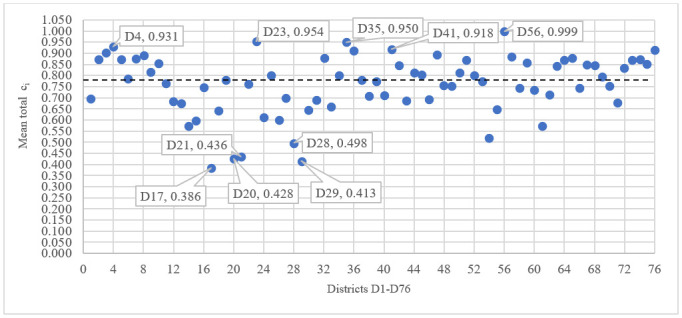
Total results of the multi-criteria evaluation of the districts’ spatial accessibility.

The total result of the multi-criteria evaluation of spatial accessibility is a mean value of *c*_*i*_ for all four indicators *(Mean total) MTc*_*i (X4*, *X5*, *X6*, *X7)*_. The values of MT*c*_*i*_ for all 76 districts are captured in the chart in Fig 8. The best five districts are: D56 Brno—City, D23 Plzeň –City, D35 Teplice, D4 Kolín and D41 Hradec Králové.

The worst five districts are: D17 Prachatice, D29 Sokolov, D20 Domažlice, D21 Klatovy and D28 Karlovy Vary. The median of the set is 0.780 (see the dashed line in the chart in Fig 8). The values of MT*c*_*i*_ and the ranking of all 76 districts are listed in the appendix.

The analysis of total and partial *c*_*i*_ values shows that the best districts from the perspective of accessibility are not those with the greatest numbers of persons over the age of 65 with lowered self-sufficiency, and vice versa, the worst accessibility is not in those districts with the lowest numbers of persons over the age of 65 with lowered self-sufficiency. The only exceptions are the Brno—City District, which has the best spatial accessibility for all types of care and transport while also having the greatest number of persons over the age of 65 with lowered self-sufficiency, and the Prachatice District, which belongs to the group of districts with the worst accessibility while also having one of the lowest numbers of persons over the age of 65 with lowered self-sufficiency.

### 4.3 The results of the correlation analysis of the indicators X1 –X7

The correlation is studied using seven indicators (X1 –X7), and the confidence interval (CI) for the correlation coefficient calculated using the bootstrap method is included. The results of the correlation—values of the correlation coefficient *r*–are listed in the matrix in Table 6.

**Table 6:** Correlation matrix.

			X1	X2	X3	X4	X5	X6	X7
X1	R		1	0.642**	0.416**	0.172	0.272**	0.370**	0.272*
	Bootstrap CI 95%	Lower		0.494	0.103	0.167	0.075	0.249	0.011
		Upper		0.750	0.476	0.298	0.416	0.527	0.416
X2	R		0.642**	1	0.244*	0.150	0.322**	0.348**	0.257**
	Bootstrap CI 95%	Lower	0.494		-0.015	0.002	0.212	0.103	0.011
		Upper	0.750		0.430	0.267	0.471	0.546	0.466
X3	R		0.416**	0.244*	1	0.162	0.259*	0.411**	0.446**
	Bootstrap CI 95%	Lower	0.103	-0.015		-0.028	0.112	0.201	0.268
		Upper	0.476	0.430		0.275	0.387	0.564	0.574
X4	R		0.172	0.150	0.162	1	0.679**	0.092	0.038
	Bootstrap CI 95%	Lower	0.167	0.002	-0.028		0.301	-0.032	-0.121
		Upper	0.298	0.267	0.275		0.884	0.490	0.435
X5	R		0.272**	0.322**	0.259*	0.679**	1	0.197	0.322**
	Bootstrap CI 95%	Lower	0.075	0.212	0.112	0.301		0.057	0.142
		Upper	0.416	0.471	0.387	0.884		0.512	0.642
X6	R		0.370**	0.348**	0.411**	0.092	0.197	1	0.503**
	Bootstrap CI 95%	Lower	0.249	0.103	0.201	-0.032	0.057		0.272
		Upper	0.527	0.546	0.564	0.490	0.512		0.709
X7	R		0.272*	0.257**	0.446**	0.038	0.322**	0.503**	1
	Bootstrap CI 95%	Lower	0.011	0.011	0.268	-0.121	0.142	0.272	
		Upper	0.416	0.466	0.574	0.435	0.642	0.709	

**Correlation is significant at the 0.01 level;

*Correlation is significant at the 0.05 level.

The interpretation of the correlation coefficient’s values is done according to [61] who determined a scale consisting of six intervals for the relationship expressed via the correlation coefficient. Within the context of the results for the calculation of *r* obtained, mostly two intervals are relevant—substantial correlation for *r* within the interval of 0.50–0.69, and a moderate correlation for *r* within the interval of 0.30–0.49.

The correlation matrix shows positive correlation relationships at the significance levels of 0.01 and 0.05 which means that when the value of one attribute (indicator) rises, so does the value of the other attribute and vice versa.

A substantial correlation relationship was detected between the following couples of indicators: (X1 and X2, *r* = 0.642); (X4 and X5, *r* = 0.679); (X6 and X7, *r* = 0.503). Therefore, it can be stated that when the number of recipients of the allowance for care (X1) rises, so does the capacity of residential services in the given area (X2) and vice versa. When the level of the spatial accessibility of residential social services via the means of individual transport improves, the level of the spatial accessibility of residential services via the means of public transport improves as well. Similarly, when the level of the spatial accessibility of the outpatient-clinic services via the means of individual transport improves, so does the level of the spatial accessibility of the outpatient-clinic services via the means of public transport.

A moderate correlation relationship was detected between the following indicators: (X1 and X3, *r* = 0.416); (X3 and X7, *r* = 0.446); (X3 and X6, *r* = 0.411). Thus, it can be stated that when the number of recipients of the allowance for care (X1) rises, so does the capacity of the outpatient-clinic services (X3), and vice versa. The capacity of the outpatient-clinic services is also significantly correlated with the level of the spatial accessibility of the outpatient-clinic services via the means of individual as well as public transport. Other interesting findings are the facts that:

- with an increase in the number of recipients of the allowance (X1), the level of the spatial accessibility of residential and outpatient-clinic services (X4 –X5) in the district **does not rise** significantly
- with an increase in the capacity of residential services (X2), the level of the spatial accessibility of residential and outpatient-clinic services (X4 –X5) **does not rise** significantly, nor does the capacity of the outpatient-clinic services (X3) in the district.

## 5 Conclusion

The results of the research bring original knowledge about the spatial offering of three types of social services intended for persons aged 65+ with reduced self-sufficiency at the level of 76 districts of the Czech Republic. The originality of the study can be seen also in the methodological concept of the evaluation of spatial accessibility gaps, which uses, among other things, the capacity of residential and outpatient services and the number of potential clients of services. Previous studies (see Literature Review) have dealt mainly with the issue of residential care and did not consider outpatient care and field care. Currently, there is no study available in the Czech Republic that focuses on the supply of social services within the context of the needs of persons with lowered self-sufficiency. Usually, only the persons’ age and the capacity of services are studied, [13,14]. It is also known, and this research confirms, that the capacity of facilities providing residential and outpatient-clinic social services is insufficient in relation to the demand in the Czech Republic [18]. The performed study, similarly to other studies (see Literature review), evaluates the spatial availability of facilities for the elderly from the point of view of defined time intervals through the GIS system.

Nevertheless, the results of this research also corroborate that besides the insufficient capacity, the facilities of residential and outpatient-clinic services are not optimally located in space. The nursing infrastructure shows a number of gaps in spatial accessibility, especially within the outpatient-clinic services. The gaps in the spatial accessibility of the outpatient-clinic services are caused by the absence of these services in some of the districts as well as by the inability to reach such services within 60 min by means of public or individual transport. There is a critical situation for persons over the age of 65 in most districts of the South Bohemian Region, the Region of Pilsen, and the Region of Karlovy Vary.

The ranking of the districts, especially in relation to the first (best) and last (worst) five, shows that the best districts do not include those with the greatest numbers of persons over the age of 65 with lowered self-sufficiency and the worst districts are not those with the lowest numbers of such persons. An exception is represented by the Brno—City District, which is the largest district in the Czech Republic in regard to the number of citizens while having the largest number of persons over the age of 65 with lowered self-sufficiency and the highest level of spatial accessibility of residential and outpatient-clinic services via the means of individual as well as public transport.

With the rising number of persons over the age of 65 with lowered self-sufficiency in the district, the capacity of residential services increased. However, this does not apply to outpatient services. The occurrence and size of gaps in spatial accessibility are influenced by individual and public transport. The results show that if the availability of services improves through individual transport, then the accessibility also improves through public transport. However, with the growth of service capacity, their spatial availability does not improve, and spatial gaps remain. This means that in most cases, no new services are set up in other municipalities in the district, but existing facilities in municipalities where services are already available are strengthened. Therefore, ineffective implementation of the National Action Plan Supporting Positive Aging in the Czech Republic can be pointed out and further discussed. This especially applies to the spatial accessibility of outpatient-clinic care for persons over the age of 65 with lowered self-sufficiency.

A future study may extend the existing study to other social services for the elderly. For example, weekly hospital appointments, sheltered housing, nursing services, and personal assistance.
